# *Poria cocos* Modulates Th1/Th2 Response and Attenuates Airway Inflammation in an Ovalbumin-Sensitized Mouse Allergic Asthma Model

**DOI:** 10.3390/life11050372

**Published:** 2021-04-21

**Authors:** Chien-Liang Chao, Chao-Jih Wang, Hsin-Wen Huang, Han-Peng Kuo, Muh-Hwan Su, Hang-Ching Lin, Chia-Wen Teng, Leticia B. Sy, Wen-Mein Wu

**Affiliations:** 1 Sinphar Pharmaceutical Co., Ltd., Sinphar Group, Yilan 269, Taiwan; chaokmc@sinphar.com.tw (C.-L.C.); zrwang@sinphar.com.tw (C.-J.W.); HWHuang@sinphar.com.tw (H.-W.H.); HPKuo1@syncorebio.com (H.-P.K.); MHSu@syncorebio.com (M.-H.S.); lhc@sinphar.com.tw (H.-C.L.); 2 Sinphar Tian-Li Pharmaceutical Co., Ltd., Sinphar Group, Hangzhou 311100, China; 3 SynCore Biotechnology Co., Ltd., Sinphar Group, Yilan 269, Taiwan; 4 School of Pharmacy, National Defense Medical Center, Taipei 114, Taiwan; 5 Department of Nutritional Science, Fu-Jen Catholic University, Hsinchuang 24205, Taiwan; CWT@mail.fju.edu.tw; 6 Cardinal Tien Hospital, Taipei 231, Taiwan; cthimsc@cth.org.tw

**Keywords:** *Poria cocos*, allergic asthma, inflammation, Th1, Th2, IgE

## Abstract

*Poria cocos*, called fuling, is a famous tonic in traditional Chinese medicine that reportedly possesses various pharmacological properties, including anti-inflammation and immunomodulation. However, few studies have investigated the effects of *P. cocos* on allergic diseases, such as allergic asthma. Allergic asthma is caused primarily by Th2 immune response and characterized by airway inflammation. This study first demonstrated the anti-allergic and anti-asthmatic effects of *P. cocos* extract (Lipucan^®^). *P. cocos* extract distinctly exhibited reduced inflammatory cell infiltration in the peribronchial and peribronchiolar regions compared to the asthma group in the histological analysis of pulmonary tissue sections. Prolonged *P. cocos* extract administration significantly reduced eosinophil infiltration, PGE2 levels, total IgE, and OVA-specific IgE. Moreover, *P. cocos* extract markedly suppressed Th2 cytokines, IL-4, IL-5, and IL-10. On the other hand, *P. cocos* extract significantly elevated IL-2 secretion by Th1 immune response. In addition, *P. cocos* extract elevated the IFN-γ level at a lower dose. We also observed that *P. cocos* extract increased the activity of NK cells. Our results suggest that *P. cocos* extract remodels the intrinsic Th1/Th2 response to prevent or alleviate allergy-induced asthma or symptoms.

## 1. Introduction

Allergic diseases are a group of disorders caused by aberrant immune responses to harmless allergens and are increasingly prevalent worldwide [1,2]. Allergic rhinitis, atopic dermatitis, food allergy, and allergic asthma are the most common allergic disorders. The molecular and cellular mechanisms of allergic diseases implicate the importance of appropriate and balanced immune response towards environmental substances [3].

Allergic diseases are closely associated with dysregulated T helper 2 cells (Th2 cells) immune responses to allergens, which are characterized by elevated secretion of Th2-specific cytokines including interleukin 4 (IL-4), IL-5, and IL-13 [4]. T helper cells (Th cells) have been suggested to act as a critical factor in allergic inflammatory response, especially Th2 cells. When encountering allergens, stimulated airway epithelial cells activate Th2 cells and release specific cytokines which in turn promote the release of other Th cytokines that expand the inflammatory response. Moreover, Th2-secreted cytokines (IL-4, IL-5, and IL-13) further trigger a series of immune responses including eosinophilic inflammation, immunoglobulin E (IgE) expression, goblet cell hyperplasia, and airway mucus secretion [5], suggested as the most striking feature of allergic disorders, including allergic asthma [6,7]. Allergic asthma is a common allergic disease and is usually characterized by variable and recurring airway symptoms, including reversible airflow obstruction in association with airway hyper-responsiveness (AHR), airway inflammation, and remodeling [8]. Allergic asthma can be triggered by exposure to various allergens such as air pollutants, chemicals, or food additives [9].

*Poria cocos* (Schwein.) F.A. Wolf (syn. *Wolfiporiacocos*), a saprophytic fungus, has been widely used as a traditional herbal medicine and its extracts have been reported to have a variety of biological activities including anti-hyperglycemic, anti-cancer, anti-inflammatory and immunomodulatory effects [10]. Two major groups of compounds have been isolated and identified from *P. cocos*, the triterpenes and the polysaccharides [11]. Several studies indicate that the anti-inflammatory activity of *P. cocos* is primarily mediated by triterpene-enriched fractions and the immunomodulatory activity mainly relies on polysaccharide-enriched fractions. Although *P. cocos* has been reported to exhibit both anti-inflammatory [12,13,14] and immunomodulatory properties [15,16,17], the effect of *P. cocos* on allergic asthma is poorly understood. In Asia, the sclerotium of *P. cocos* is a common edible medicinal fungus, known as “Fuling” in Chinese, which is usually in the form of dry powder or capsules on the market. Moreover, Fuling is not only used as a treatment for diseases but also as a daily supplement to improve or enhance health [18,19]. Furthermore, our previous study demonstrated that *P. cocos* extract (Lipucan^®^) containing lanostane triterpenoids plays beneficial roles in immunoregulatory activity [20].

The clinical symptoms may be different according to the affected organs and even patients, but all allergic disorders have a similar pathological mechanism resulting from dysregulated immune response and subsequent harmful inflammation. Since allergic diseases are intrinsically caused by dysregulated immune response, the modulation of immune response by complementary and alternative medicine may provide another way to improve the situation of patients. Here, a commercial capsule, which contains 27 mg of *P. cocos* extract (Lipucan^®^), was used to investigate the effects of *P. cocos* on the improvement of allergic disorders, including allergic asthma. Additionally, an ovalbumin (OVA)-induced airway hypersensitivity mouse model was used as a tool to study allergic asthma. Our OVA-sensitized asthma mouse model included two parts of OVA sensitization: chronic OVA sensitization by intraperitoneal (i.p.) injection three times (with10-days intervals) and acute airway sensitization by acute OVA inhalation. By using this OVA-sensitized asthma mouse model, we could evaluate the *P. cocos* extract’s effects on OVA-induced chronic inflammation and acute allergic asthma responses. OVA-sensitized mice have been reported to exhibit elevated leukocyte infiltration into the airways, particularly eosinophils, which secrete specific chemokines, such as eotaxin, as well as cytokines, such as IL-4 [21,22]. Our results showed that prolonged *P. cocos* extract administration significantly alleviated eosinophil infiltration into airways and airway inflammation, and modulated Th1 and/or Th2 immune responses, which are critical for asthma induction. Moreover, we also found that prolonged *P. cocos* extract administration enhanced natural killer (NK) cell activity.

## 2. Materials and Methods

### 2.1. Poria cocos Extract (Lipucan^®^)

**Figure 1:**
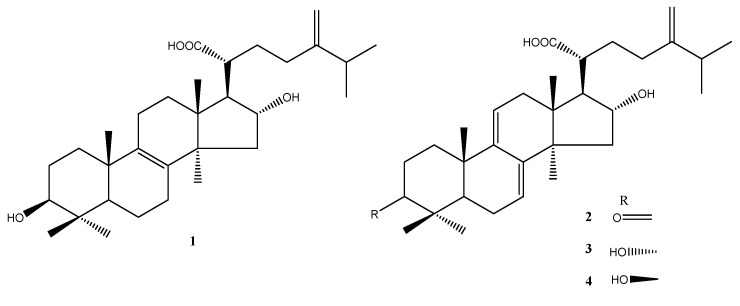
The chemical structures of lanostane triterpenoids **1**–**4** isolated from *P. cocos*.

The material consisted of *P. cocos* extract, named Lipucan^®^, which was manufactured by Sinphar Tian-Li Pharmaceutical Co, Ltd (Hangzhou, China), Hangzhou Sinphar Group in China and developed by Sinphar R&D Center, Taiwan (Yilan, Taiwan). Four major lanostane triterpernoids (tumulosic acid (**1**), polyporenic acid C (**2**), 3-epi-dehydrotumulosic acid (**3**), and dehydrotumulosic acid (**4**)) (Figure 1) make up *P. cocos* extract (Lipucan^®^) content at 6.2%. In this study, a commercial capsule, containing 27 mg of *P. cocos* extract (Lipucan^®^), was prepared to investigate the effects of *P. cocos* on OVA-sensitized allergic mice.

### 2.2. Animals

Inbred BALB/c mice (6–8 weeks old and weighed 15–20 g) were obtained from BioLASCO (Taipei, Taiwan) and housed at the Department of Nutrition and Food Sciences, Fu Jen Catholic University. Mice were maintained under specific pathogen-free conditions and given free access to sterile water and chow. Animal experiments were performed according to the Guidelines for the Care and the Use of Laboratory Animals, FuJen University. The mice were randomly divided into five groups. Each group consisted of 10 mice, namely, the asthmatic group, *P. cocos* extract-administrated group (FL-800, FL-1200, and FL-1600), and prednisolone group.

### 2.3. Establishment of the Experimental Animal Model of Asthma and Treatments

**Figure 2:**
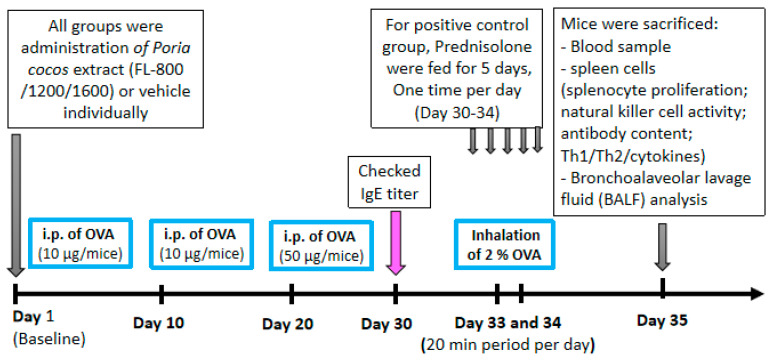
Schematic representation of the protocol for ovalbumin-induced airway inflammation in mice, and treatment with *P. cocos* extract or prednisolone (treated at day 30–34).

The immunization protocol (Figure 2) was similar to that described previously and modified [20,23]. BALB/c mice were administered with or without *Poria cocos* extracts daily by tube feeding that started at 8 weeks of age and continued up to the day of sacrifice (day 35). Mice were sensitized by i.p. injection of 10, 10, and 50 μg OVA (Sigma Chemical Co., St Louis, MO, USA) in phosphate-buffered saline (PBS) combined with the adjuvant aluminum hydroxide (Pierce) at day 1, 10, and 20, respectively. On day 33 and 34, mice were challenged with aerosol of OVA (2%) for 20 min in a closed chamber connected to a nebulizer (DeVibissPulmo-Aide, 5650D, Port Washington, New York, NY, USA), under a continuous aerosol flow. Every time after OVA sensitization, the mice were bled via the retro-orbital plexus for OVA-specific IgE using ELISA to confirm the allergic asthma induction. OVA-sensitized mice were orally treated with vehicle, *P. cocos* extract (104 mg/kg (FL-800), 156 mg/kg (FL-1200), and 208 mg/kg (FL-1600)), or prednisolone (5 mg/kg) as the positive control. *P. cocos* extract groups were administrated for 5 days/week for 5 weeks. The prednisolone group was only administrated with prednisolone (5 mg/kg) daily by tube feeding 5 days before sacrifice.

### 2.4. BronchoalveolarLavage Fluid (BALF) Analysis and Histological Analysis

After the last OVA inhalation challenge, the mice were sacrificed and the lungs were immediately lavaged via the trachea cannula with 1mL of Hank’s balanced salt solution (HBSS, Atlanta Biologics, Norcross, GA, USA). The BALF was centrifuged at 1500 rpm for 5 min at 4 °C and supernatants were kept at −20 °C until analysis for eotaxin, IL-5, LTC4, and PGE2. BALF cells were resuspended in culture medium (100 μL) and centrifuged (Cytospin 4 cytocentrifuge, ThermoShadon, Waltham, MA, USA) at 500 rpm for 5 min, and stained with Liu’s stain solution (Liu A and Liu B, Delta, Tokyo, Japan). The number of each cell type (eosinophils, lymphocytes, neutrophils, and monocytes) was determined by morphological criteria and 200 leukocytes were counted on each slide. To evaluate the *P. cocos* extract’s effects on lung inflammation, the lungs were excised intact, after BALF collection, and then perfused with 4 % formalin. Sections (5 µm thick) were stained with hematoxylin–eosin and examined for histological changes using a light microscope (Olympus, BX51, Tokyo, Japan).

### 2.5. Determination of Eotaxin, IL-5, LTC4, and PGE2 in BALF

Eotaxin, IL-5, LTC4, and PGE2 levels in BALF were determined using an ELISA assay according to the manufacturer’s instruction (eotaxin by R&D, Duoset, Minneapolis, MN, USA, IL-5 by Pharmigen, San Diego, CA, USA, LTC4 by Cayman, Ann Arbor, MI, USA, and PGE2 by Cayman, USA). Briefly, the BALFs were added to the wells which were coated with specific monoclonal antibodies overnight at 4 °C, and incubated at room temperature for 2 h. After 2 h of incubation, the plates were washed and biotin-conjugated antibodies were added for an additional 2 h at room temperature. HRP-avidin was then added to each well for 2 h. The substrate tetramethylbenzidine was then added and the OD (at 450 nm) values were measured and calculated to estimate the cytokine concentrations.

### 2.6. Determination of Total IgE, IgA, IgM, IgG, and OVA-Specific IgE

Levels of total IgE, IgA, IgM, IgG, and OVA-specific IgE in serum were determined by a capture ELISA assay (BETHYL, Montgomery, TX, USA) as described previously [24] with slight modifications. ELISA plates were coated with IgE, IgA, IgM, or IgG antibody for 1 h at room temperature. Then, the plate was washed with PBS and blocked with 1% bovine serum albumin for 1 h at room temperature. After blocking, the plate was washed 6 times with Tris-buffer (pH 8.0) and standard or sample was added for 1 h at room temperature. After washing, the substrate tetramethylbenzidine was added and the absorbance at 450 nm was measured using an ELISA reader (Dynatech laboratories MRX2CXB-2523, Chantilly, VA, USA). For OVA-specific IgE, ELISA plates were coated with 200 μg/mL of OVA in 0.1 M NaHCO_3_ (pH 9.6) and incubated overnight at 4 °C. Then, the plate was washed and further blocked with 1% bovine serum albumin for 2 h at room temperature. Samples or standard were added into wells and incubated at 4 °C overnight. The plates were then washed and incubated with HRP-conjugated anti-mouseIgE antibody overnight at 4 °C. 

### 2.7. Cytokine Levels in Splenocytesand BALF Cultured Supernatants

After mice were sacrificed, the spleens were aseptically removed and crushed into a single cell suspension. Red blood cells were lysed with Tris-buffered ammonium chloride. Cells were then washed three times with Hank’s balanced salts solution. To evaluate the Th1 and Th2 responses in OVA-sensitized mice administrated with or without *P. cocos* extract, splenocytes (1 × 10^6^ cells/mL) were treated with either 5 μg/mL concanavalin A (ConA) or 100 μg/mL OVA for 48 h and cytokines such as IL-2, IL-4, IL-5, IL-10, and IFN-γ in the supernatants were determined using an ELISA assay. Eotaxin, IL-2, IL-4, IL-5, IL-10, and IFN-γ levels were determined using an ELISA assay according to the manufacturer’s instructions.

### 2.8. Evaluation of NK Cell Activity

In order to evaluate the NK cell activity, we used a LIVE/DEAD Cell-Mediated Cytotoxicity kit (Molecular Probes) according to the manufacturer’s instructions. Briefly, the splenocytes (2 × 10^6^ cells/mL, as a kind of effector cell) collected from OVA-sensitized mice were individually co-cultured with YAC-1 cells (1 × 10^4^ cells/mL). YAC-1 cells werestained by green fluorescent dye DioC18 and actedas target cells which may be attacked by the NK cells in splenocytes. The effector/target (ET) ratio of splenocytes to YAC-1 was 200:1. After 4 hours of incubation, dead YAC-1 cells were stained with propidium iodide (PI) according the protocol of the LIVE/DEAD Cell-Mediated Cytotoxicity kit (Molecular Probes, L-7010) and analyzed by flow cytometry (EPICS XL-MLC, Beckman Coulter, Brea, CA, USA). Collected data were analyzed by fluorescence intensity with WinMDI 2.8 software to estimate the cytotoxic activity of the splenocytes [20].

### 2.9. Statistics

Data were expressed as mean ± SD and analyzed by one-way ANOVA followed by Dunnett’s test. *p* < 0.05 was considered as statistically significant. 

## 3. Results

### 3.1. P. cocos Reduced Inflammation in BALF and Lung Tissues in OVA-Sensitized Mice

**Figure 3:**
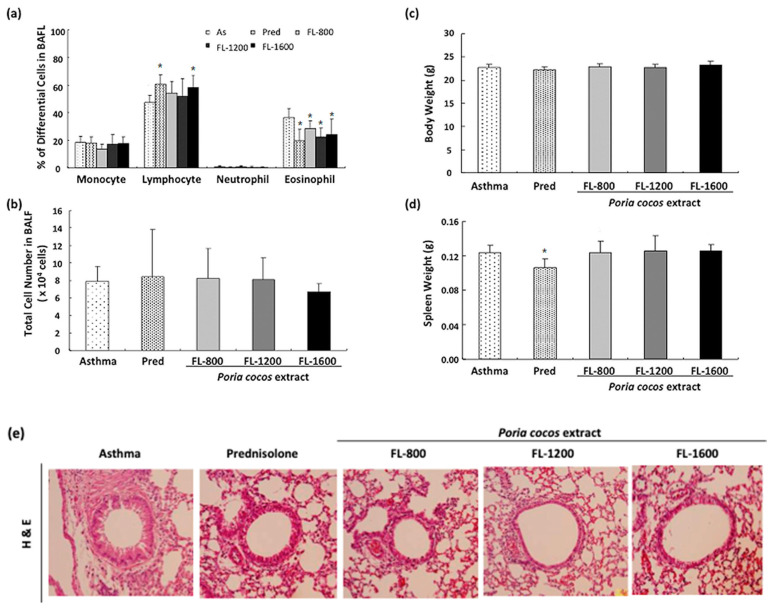
*P. cocos* extract effect on airway inflammation in OVA-sensitized mice. OVA-sensitized mice were administrated with a vehicle, *P. cocos* extract, or prednisolone (positive control). The experimental and control groups of mice were sacrificed and analyzed for percentages of differential white blood cells (**a**) or total cell number (**b**) in bronchoalveolar lavage fluid (BALF). Mouse body weights (**c**) were measured before sacrifice and spleen weights (**d**) were weighed after sacrifice. The mean and SD values are shown for the five groups (asthma (As), prednisolone (Pred), FL-800, FL-1200, and FL-1600) of OVA-sensitized mice. Each group contained 10 mice. Data were analyzed statistically using one-way ANOVA and Dunnett’s test. * *p* < 0.05 compared with the asthma group. Histological analysis of lung sections (**e**). Mouse lung sections (6 mm) were stained with H&E (×200).

First, to assess whether prolonged *P. cocos* extract administration alleviates the airway inflammation, the inflammatory cell composition of BALF from OVA-sensitized mice administrated with PBS or different *P. cocos* extract doses (FL-800, FL-1200, FL-1600), or prednisolone (as a positive control) was analyzed. As shown in Figure 3a, *P. cocos* extract administration significantly reduced eosinophil infiltration at all doses, compared to the asthma group. Moreover, the FL-1200 and FL-1600 doses exhibited similar effects on eosinophil reduction in BALF to the prednisolone positive control group. On the other hand, the percentage of lymphocytes was significantly higher in the BALF from both OVA-sensitized mice administrated with the FL-1600 dose or prednisolone than from asthmatic mice. There was no difference in the total cell number between the control and drug-administrated groups (Figure 3b). In addition, *P. cocos* extract administration had no effect on body weights (Figure 3c) or spleen weights (Figure 3d) of OVA-sensitize mice, while prednisolone treatment led to a decrease in spleen weight but not body weight, compared to the asthma group.

Histological analysis of the pulmonary tissue sections revealed that all doses of *P. cocos* extract exhibited reduced inflammatory cell infiltration in the peribronchial and peribronchiolar regions compared to the asthma group (Figure 3e). The prednisolone group also showed no obvious cell infiltration compared to the asthma group. Together, these results suggest that *P. cocos* extract administration alleviated airway inflammation in bronchoalveolar lavage fluid (BALF) and lung tissues in OVA-sensitized mice. 

### 3.2. P. cocos Suppressed the Release of Total IgE in Blood Serum

**Figure 4:**
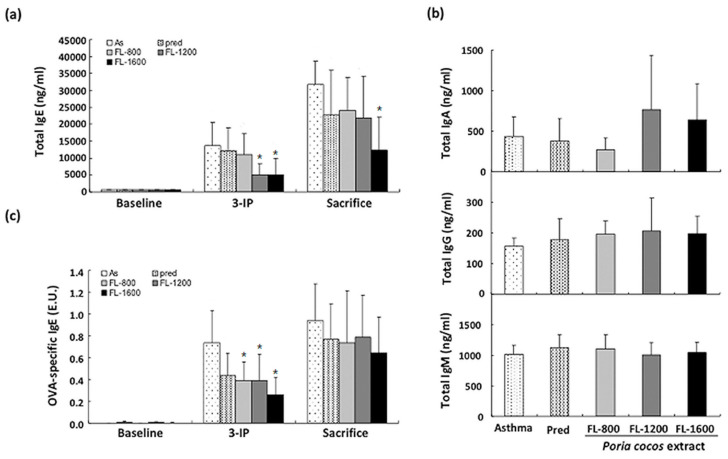
*P. cocos* extract effect on serum total IgE, IgM, IgG, IgA, and OVA-specific IgE levels in OVA-sensitized mice. The total IgE (**a**), IgM, IgG, IgA (**b**), and OVA-specific IgE (**c**) levels were determined using ELISA. 3-IP in (**a**) and (**c**) mean that mice were sensitized by i.p. injection of OVA for three times at day 1, 10, and 20. The mean and SD values are shown for the five groups (asthma (As), prednisolone (Pred), FL-800, FL-1200, and FL-1600) of OVA-sensitized mice. Each group contains 10 mice. Data were analyzed statistically using one-way ANOVA and Dunnett’s test. * *p* < 0.05 compared with the asthma group.

Elevated serum total IgE level in asthmatic patients is highly correlated with an increase in Th2 cytokines [8,25,26]. Therefore, we next investigated the *P. cocos* extract’s effects on the serum IgE concentration in OVA-sensitized mice. Interestingly, we found that there were differential *P. cocos* extract effects on serum IgE concentration by the time points of the collection of blood. As shown in Figure 4a, there is no significant difference in serum total IgE concentration between asthma and drug-administrated groups before OVA sensitization. After the last OVA i.p. injection, the serum IgE concentration was significantly decreased in FL-1200 and FL-1600 groups, while no significant changes occurred in groups with FL-800 or prednisolone treatment. To further investigate the long-term *P. cocos* extract administration effect on allergic airway inflammation, sensitized mice were acutely challenged with OVA inhalation to induce airway inflammation and the serum total IgE concentration was evaluated. Notably, FL-1600, the highest dose, even significantly reduced the serum IgE titer in mice sensitized with the OVA inhalation procedure before sacrifice, compared to that in asthma group (Figure 3a). On the other hand, there are no significant changes in total serum IgM, IgG, and IgA between control and drug-administrated groups (Figure 4b). These results suggest that *P. cocos* extract suppressed total IgE secretion in OVA-sensitized mice. 

OVA-induced specific IgE was reported to be associated with inflammatory cells in allergic asthma pathogenesis [27]. We also examined whether *P. cocos* extract affects the OVA-specific IgE level in OVA-sensitized asthmatic mice. After the last OVA i.p. injection, OVA-specific IgE was significantly increased, compared to baseline (Figure 4c). *P. cocos* extract administration significantly reduced the OVA-specific IgE titer at all doses while prednisolone treatment had only a marginal effect on OVA-specific IgE attenuation. However, *P. cocos* extract had no effect on OVA-specific IgE titer attenuation in sensitized mice with ovalbumin inhalation before sacrifice, as well as prednisolone treatment. 

### 3.3. P. cocos Suppressed OVA-Induced Chemokine and Cytokine Secretion in BALF

**Figure 5:**
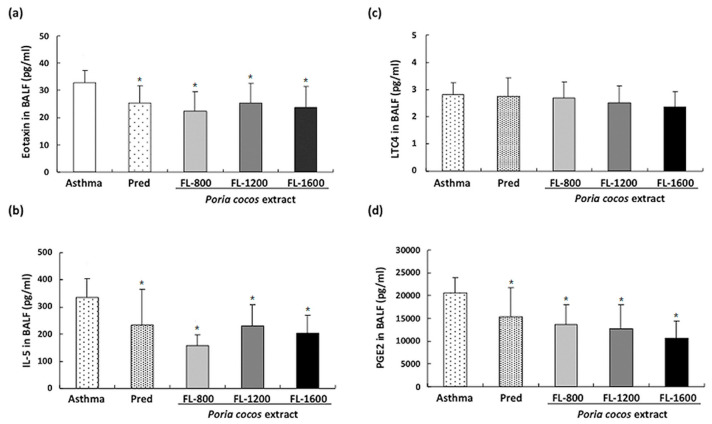
Effects of *P. cocos* extract on cytokine and chemokine levels in the BALF of OVA-sensitized mice. The eotaxin (**a**), IL-5 (**b**), LTC4 (**c**), and PGE2 (**d**) levels in BALF of OVA-sensitized mice were analyzed using ELISA. The mean and SD values are shown for the five groups (asthma, prednisolone (Pred), FL-800, FL-1200, and FL-1600) of OVA-sensitized mice. Each group contains 10 mice. Data were analyzed statistically using one-way ANOVA and Dunnett’s test. * *p* < 0.05 compared with the asthma group.

The increased IgE concentration in OVA-sensitized mice indicates that the elicited Th2 response involves IL-4, IL-5, and IL-13 [28]. Moreover, elevated leukocyte translation into the airways, particularly eosinophils, is a critical phenomenon in the OVA-induced asthma mouse model, correlated with specific chemokines, such as eotaxin [21], and also regulated by Th2-secreted cytokines such as IL-5 [22]. To investigate whether prolonged *P. cocos* extract administration affects the OVA-induced Th2-predominant immune reaction in the lungs, levels of Th2-type cytokines, IL5 and eotaxin, in BALF were analyzed. *P. cocos* extract or prednisolone administration in OVA-sensitized mice significantly reduced the eotaxin concentrations (Figure 5a) and IL-5 (Figure 5b), compared to the asthma group. To further examine the *P. cocos* extract effect on airway inflammation attenuation, we next examined leukotriene C4 (LTC4) (Figure 5c) and prostaglandin E2 (PGE2) (Figure 5d), two important lipid mediators which have been implicated in eosinophil-associated inflammation pathogenesis in bronchial asthma [29]. *P. cocos* extract administration significantly reduced the PGE2 level but had little to no effect on the LCT4 level in the BALF of mice sensitized and challenged with OVA compared to the asthma group. 

### 3.4. P. cocos Effects on Cytokine Release in ConA-Stimulated Cultured Splenocytes Derived from OVA-Sensitized Mice

**Figure 6:**
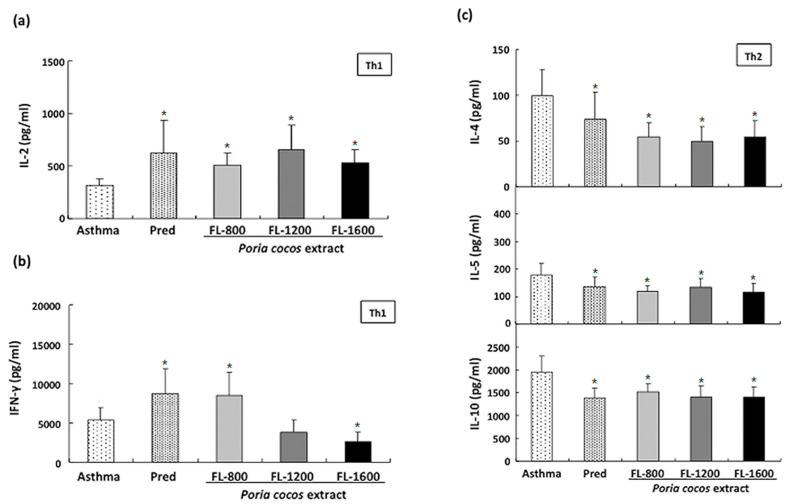
*P. cocos* extract effects on Th1/Th2 cytokine secretion by ConA-stimulated cultured splenocytes derived from OVA-sensitized mice. Th1-specific cytokine, IL-2 (**a**) and IFN-γ (**b**), andTh2-specific cytokine, IL-4, IL-5, and IL-10 (**c**), levels in splenocyte culture supernatants derived from OVA-sensitized mice with different treatments were determined by ELISA. The mean and SD values are shown for the five groups (asthma, prednisolone (Pred), FL-800, FL-1200, and FL-1600) of OVA-sensitizedmice. Each group contains 10 mice. Data were analyzed statistically using one-way ANOVA and Dunnett’s test. * *p* < 0.05 compared with the asthma group.

The Th1/Th2 cytokine production imbalance has been implicated as a critical factor in patients with allergic asthma [30]. To further investigate the *P. cocos* extract’s effect on the Th1/Th2 responses, Th1-specific cytokine (IFN-γ and IL-2) and Th2-specific cytokine (IL-4, IL-5, and IL-10) levels in the supernatant of cultured splenocytes derived from OVA-sensitized mice in response to ConA were analyzed. As shown in Figure 6a, in ConA-stimulated splenocyte supernatant, all *P. cocos* extract doses exhibited a significant increase in IL-2 level, compared to that in the asthma group. On the other hand, ConA-stimulated splenocytes derived from OVA-sensitized mice administrated with *P. cocos* extract showed a biphasic effect on IFN-γ level in supernatants, compared to cells from the asthma control group (Figure 6b). FL-800, the lowest dose, similar to the prednisolone group, significantly increased IFN-γ level in ConA-stimulated splenocytes supernatants. In contrast, OVA-sensitized mice administrated with FL-1600, the highest dose, exhibited a lower supernatant IFN-γ level than OVA-sensitized mice without drugs. Moreover, Th2-specific cytokines levels, including IL-4, IL-5, and IL-10, were lower in *P. cocos* extract-administrated groups at all doses than those in the asthma control group (Figure 6c). Similarly, the prednisolone positive control group also showed reduced levels of IL-4, IL-5, and IL-10 compared to the asthma group. 

### 3.5. P. cocos Effects on Cytokine Release in OVA-Stimulated Cultured Splenocytes Derived from OVA-Sensitized Mice

**Figure 7:**
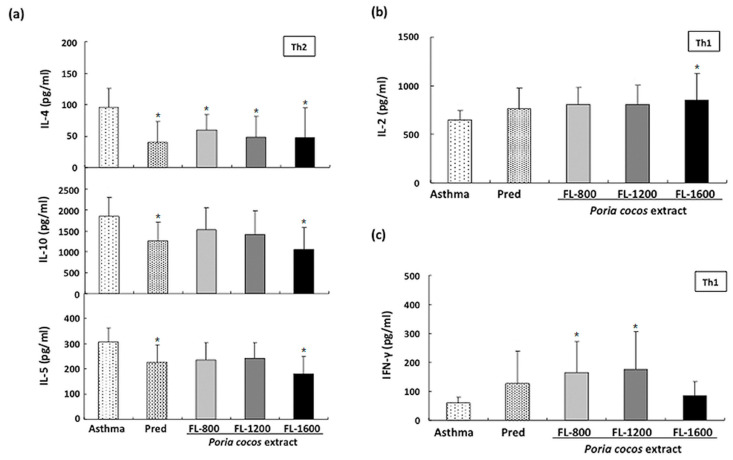
*P. cocos* extract effects on Th1/Th2 cytokine secretion by OVA antigen-stimulated cultured splenocytes derived from OVA-sensitized mice. Cultured splenocytes derived from OVA-sensitized mice were stimulated with 100 μg/ml OVA and culture supernatants were collected, and the IL-4, IL-5, IL-10 (**a**), IL-2 (**b**), and IFN-γ (**c**) levels were determined using ELISA. The mean and SD values are shown for the five groups (asthma, prednisolone (Pred), FL-800, FL-1200, and FL-1600) of OVA-sensitizedmice. Each group contains 10 mice. Data were analyzed statistically using one-way ANOVA and Dunnett’s test. * *p* < 0.05 compared with the asthma group.

To further confirm the *P. cocos* extract effect on Th1/Th2 response modulation under allergic stimulation, we examined the *P. cocos* extract’s effect on OVA-induced cytokine release in cultured splenocytes derived from OVA-sensitized mice. The IL-4, IL-5, and IL-10 levels in OVA-stimulated cultured splenocyte supernatants derived from sensitized mice administrated with *P. cocos* extract were significantly reduced, compared to those in asthmatic mice (Figure 7a), implicating that long-term *P. cocos* extract administration could modulate Th2-mediated response.

On the other hand, FL-1600 significantly increased the IL-2 level in OVA-stimulated splenocyte supernatants derived from OVA-sensitized mice, while other *P. cocos* extract and prednisolone doses only showed marginal effects (Figure 7b). Furthermore, *P. cocos* extract administration exhibited a dose-dependent biphasic effect on IFN-γ level in OVA-stimulated splenocyte supernatants (Figure 7c). FL-800 and FL-1200 significantly increased the IFN-γ level in OVA-stimulated splenocyte supernatants compared to the asthma group, while FL-1600 did not show a statistical difference to the asthma group (Figure 7c). 

### 3.6. P. cocos Effects on NK Cell Activity in OVA-Sensitized Mice 

**Figure 8:**
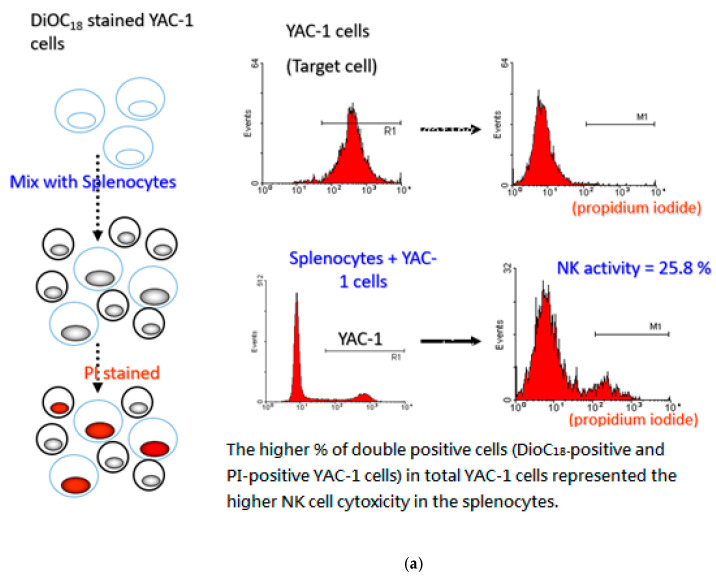

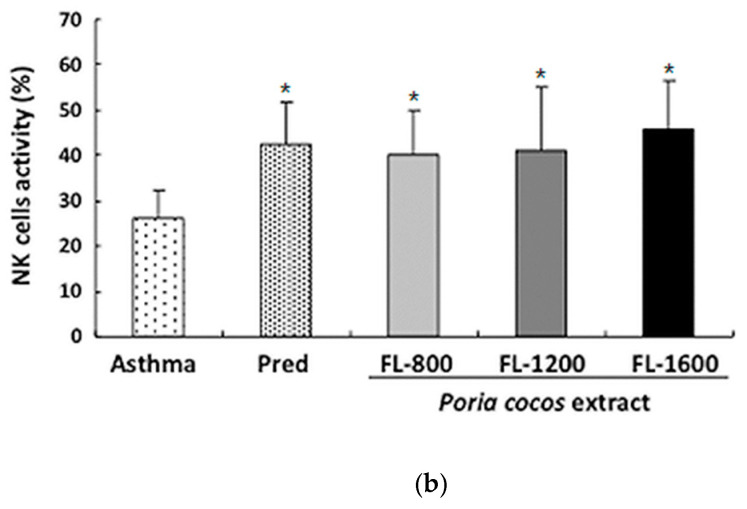
*P. cocos* extract effects on activity of NK cells derived from OVA-sensitized mice spleens. Splenocytes were isolated from OVA-sensitized mice administrated with a vehicle, *P. cocos* extract, or prednisolone and co-cultured with target YAC-1 cells for the determination of NK cell cytotoxic activity using flow cytometry. (**a**) The schematic diagram of DioC18-stained YAC-1 cells and flow cytometry of asthma group. (**b**) The NK cell activity (%). The mean and SD values are shown for the five groups (asthma, prednisolone (Pred), FL-800, FL-1200, and FL-1600) of OVA-sensitized mice. Each group contains 10 mice. Data were analyzed statistically using one-way ANOVA and Dunnett’s test. * *p* < 0.05 compared with the asthma group.

The *P. cocos* extract’s effect on NK cell activity in OVA-sensitized mice is shown in Figure 8. *P. cocos* extract and prednisolone significantly enhanced NK cells’ killing activity in the OVA-sensitized mice. 

## 4. Discussion

For a long time in many areas of Asia, especially in China, medicinal herbs have been widely used as ingredients of traditional medicines for various diseases and also as supplements for health improvement in many ways. *P. coco* is an herbal remedy commonly used in traditional Chinese medicine for a range of medical or health conditions. Notably, some studies showed that the composition of *P. cocos* exhibits anti-inflammatory activity and immunomodulatory properties [10,12,13,14,15,16,17,20], implying apotential role for *P. cocos* extract in the treatment of allergic asthma, an immune dysregulation disease. In this study, we evaluated the therapeutic effects of *P. cocos* extract in OVA-induced animal asthma models and investigated the mechanism by which *P. cocos* extract affects the Th1/Th2 response in allergic asthma. 

We found that prednisolone treatment led to a loss of spleen weight. Since prednisolone is a type of corticosteroid, prednisolone-induced loss of spleen weight may be caused by its strong suppressive activity in the immune system. Compared to the toxic effect of prednisolone treatment on spleen weight, long-term *P. cocos* extract administration showed no significant body and spleen weight loss, indicating that the *P. cocos* extract doses used here were less or not toxic for animals.

Increased serum IgE is one of the striking features of OVA-induced allergic asthma [5]. We observed that *P. cocos* extract significantly reduced total IgE serum level, while prednisolone treatment had little to no effect on it. This indicated that prolonged *P. cocos* extract administration has a better effect than prednisolone on increased IgE attenuation in asthmatic mice. Notably, we found that *P. cocos* extract exhibited better activity in reducing serum total IgE level in mice before final OVA inhalation sensitization than after OVA inhalation. Moreover, the *P. cocos* extract significantly attenuated the OVA-specific IgE level in mice before final OVA inhalation sensitization, while it had little to no effect after OVA inhalation. These results indicate that prolonged *P. cocos* extract administration showed relatively low activity on acute airway inflammation attenuation.

Typical OVA-induced allergic asthma features in mice are inflammatory cell infiltration, particularly eosinophils, IgE expression, and goblet cell hyperplasia and airway mucus secretion [5]. Our results showed that prolonged *P. cocos* extract administration significantly reduced the numbers of eosinophils, eotaxin, IL-5, and PGE2 in the BALF of asthmatic mice. This suggests that prolonged *P. cocos* extract administration could efficiently ameliorate airway inflammation in OVA-sensitized asthmatic mice. However, whether the levels of eotaxin, IL-5, and PGE2 in BALF or the reduction of eosinophil infiltration suggest that *P. cocos* extract indeed ameliorated the airway inflammation situationis Moreover, PGE2-mediated signaling has been recently reported to promote IgE levels during asthma development [31], implying that the *P. cocos* extract’s inhibitory effect on elevated PGE2 in BALF of asthmatic mice may attenuate asthma development via PGE2 suppression.

Many studies have indicated that an asthma attack is the result of imbalanced immune responses related to Th1/ Th2 cytokine production. In the lung tissue, Th2 cells can respond to inhaled allergens by secreting IL-4, IL-5, and other mediators, representing chronic airway inflammation [29]. Moreover, Th2 cell-mediated immune response to allergens has been suggested as a critical leading factor for allergic asthma [32,33,34]. Therefore, modulation of Th2 cell-mediated response to an allergen may be a good alternative strategy for allergic asthma treatment. Our results show that prolonged *P. cocos* extract administration significantly reduced Th2cytokine secretion, including IL-4, IL-5, and IL-10 in OVA- or ConA-stimulated splenocytes. However, the *P. cocos* extract’s effects on IFN-γ or IL-2, two Th1cytokines, were divergent. *P. cocos* extract significantly elevated IL-2 secretion at all doses in OVA- or ConA-stimulated splenocytes. Interestingly, *P. cocos* extract exhibited a dose-dependent biphasic effect on IFN-γ secretion, IFN-γ secretion elevation at lower doses, and a decrease or constancy of IFN-γ secretion at the highest dose of *P. cocos* extract, compared to the asthma group. Moreover, IL-2 and/or IFN-γ have been reported to activate NK cell activity [35]. Consistently, we also observed that NK cell activity was significantly increased in the *P. cocos* extract group, which led to elevated secretion of IL-2 (and IFN-γ in some doses). Interestingly, IL-2 has been reported to activate NK cell activity in the absence of IFN-γ [36]. According to our results, we suggest *P. cocos* extract may target NK cells. A robust NK cell response to respiratory viral infection with high levels of IFN-γ production and cytolytic activity leads to viral clearance and a Th1 environment in the lung and normal tolerance of environmental antigens [37]. An impaired response to viral infection with low IFN-γ and cytolytic activity and higher levels of Th2-type cytokines released from NK cells leads to increased inflammation and viral shedding, the recruitment of immature dendritic cells (DCs), and allergic sensitization with subsequent inflammation [37]. *P. cocos* extract can modulate Th1/Th2 response under allergic stimulation. This includes more Th1 cytokines (IFN-γ and IL-2) combined with fewerTh2 cytokines (IL-4 and IL-5, Figure 6 and Figure 7), correlated with significantly higher NK cell cytotoxicity as compared to the asthma group (Figure 8). Collectively, our results indicate that *P. cocos* extract was able to modulate Th1-mediated responses and had an inhibitory effect on the Th2-mediated responses.

The prevalence of allergic diseases, including allergic asthma and atopic dermatitis, has considerably increased globally in recent years [38]. Possible reasons for the increasing prevalence of allergic diseases globally may be at least in part attributable to environmental changes or impacts, such as air pollution and climate change caused by urbanization and industrialization. Growing evidence shows that atmospheric particulate matter less than 2.5 μm in diameter, known as PM2.5, may lead to allergic disorders such as airway inflammation and asthma [39,40]. All allergic diseases result from adysregulated or imbalanced immune response, mainly a Th2-mediated immune response, to harmless environmental substances, implying that immune system modulation may also facilitate other allergic disorders such as atopic dermatitis and food allergy. Currently, the therapeutic strategy for allergic disorders like allergic asthma is to directlytreat and alleviate the acute symptoms caused by immune system overreaction, facilitating relief from discomfort as much as possible. However, some patients do not respond to this treatment or have some unpleasant side effects. Therefore, prevention treatments for allergic diseases are worth being investigated. Our study addressed the basic understanding of *P. cocos* extract in the alleviation of allergic disorders including allergic asthma and provides an alternative strategy for the treatment of allergic diseases, such as asthma.

## 5. Conclusions

This study first demonstrated the anti-allergic and anti-asthmatic effects of *P. cocos* extract (Lipucan^®^). Prolonged *P. cocos* extract administration significantly reduced eosinophil infiltration, PGE2 levels, total IgE, and OVA-specific IgE. *P. cocos* extract markedly exhibited reduced inflammatory cell infiltration in the peribronchial and peribronchiolar regions compared to the asthma group in the histological analysis of the pulmonary tissue sections. In addition, *P. cocos* extract markedly suppressed Th2cytokines, IL-4, IL-5, and IL-10, secretions that have been suggested as a potential therapeutic target in allergic asthma. On the other hand, *P. cocos* extract significantly elevated IL-2 secretion. However, *P. cocos* extract exhibited a biphasic effect on the IFN-γ level in a dose-dependent manner: elevated at lower doses and decreased at the highest dose. We also observed that *P. cocos* extract increased the natural killer (NK) cell activity. Our results suggest that *P. cocos* extract exerted an anti-allergic effect and suggested an alternative strategy with long-term administration of immunomodulatory compounds, such as *P. cocos* extract, to remodel the intrinsic Th1/Th2 response to prevent or alleviate allergy-induced asthma or symptoms.

## Data Availability

The data presented in this study are available on request from the **c**orresponding authors. The data are not publicly available.
